# Lamb-Shaffer syndrome in a Chinese adolescent: A case report

**DOI:** 10.1097/MD.0000000000047820

**Published:** 2026-03-06

**Authors:** Jingting Xu, Xiao Song, Linghui Zeng, Chaochun Zou

**Affiliations:** a Department of Endocrinology, Children’s Hospital, Zhejiang University School of Medicine, Hangzhou, China; b The Clinics, Hangzhou Children’s Welfare Institute, Hangzhou, China; c Department of Clinical Medicine, Hangzhou City University School of Medicine, Hangzhou, China; d Zhejiang Key Laboratory of Neonatal Diseases, Hangzhou, China.

**Keywords:** clinical manifestation, diagnosis, management, mutation, *SOX5* gene

## Abstract

**Rationale:**

Lamb-Shaffer syndrome (LAMSHF) is a rare neurodevelopmental disorder caused by pathogenic variants in the SRY-related high-mobility group box 5 (*SOX5*) gene. Clinical features are heterogeneous, and novel variants continue to be reported, expanding the genotypic and phenotypic spectrum of the disease.

**Patient concerns:**

A 15-year-old male presented with short stature, mild intellectual disability, epilepsy, and multiple congenital anomalies, including facial dysmorphism and right thumb syndactyly.

**Diagnoses:**

Whole-exome sequencing identified a novel heterozygous variant in the *SOX5* gene, c.1160G>A (p.Ser387Asn), located at 12p12.1. Although initially classified as a variant of uncertain significance according to ACMG criteria, its strong correlation with the clinical phenotype supported the diagnosis of LAMSHF.

**Interventions:**

The patient has been maintained on levetiracetam for epilepsy management and is receiving dental care for maxillofacial deformities. A multidisciplinary rehabilitation approach is recommended.

**Outcomes:**

Seizures are well-controlled with no recurrence. The patient demonstrates stable cognitive and functional status under current supportive care.

**Lessons:**

This case reports a novel *SOX5* variant associated with LAMSHF and highlights the importance of genetic confirmation in patients with unexplained neurodevelopmental features to guide appropriate management and avoid unnecessary interventions.

## 1. Introduction

Lamb-Shaffer syndrome (LAMSHF, Online Mendelian Inheritance in Man (OMIM) 616803) is a neurodevelopmental disorder characterized by distinctive facial features, central nervous system abnormalities, and skeletal anomalies. It is caused by pathogenic variants in the SRY-related high-mobility group box 5 (*SOX5*) gene, which encodes a transcription factor essential for neural and chondrogenic development. This condition is extremely rare, with only slightly more than 100 cases reported. Its broad and variable clinical manifestations often lead to misdiagnosis or underdiagnosis. *SOX5* mutations have also been associated with several malignancies. Deletions involving the 12p12 locus have been linked to head and neck squamous cell carcinoma, pancreatic ductal adenocarcinoma, colon cancer, and cholangiocarcinoma. This potential predisposition to aggressive tumors suggests adverse prognostic implications for affected children and warrants long-term follow-up.^[1]^ Moreover, these findings guide clinical decision-making, particularly in avoiding growth hormone therapy to reduce tumorigenic risk. Herein, we present a pediatric case of LAMSHF and review the literature to expand the knowledge base on this rare disorder, focusing on its pathogenesis, clinical manifestations, and management.

## 2. Methods

### 2.1. Participant consent and ethical approval

The study involved a 15-year-old adolescent boy residing at the Hangzhou Children’s Social Welfare Center. It adhered to the CARE guidelines and complied with the principles of the Declaration of Helsinki. Ethical approval was obtained from the Ethics Committee of the Children’s Hospital of Zhejiang University School of Medicine. Informed consent for participation was obtained from the legal guardians of the patient, who also provided consent for the publication of clinical details and images.

### 2.2. Patient information

Clinical data were collected, including demographic information, physical examination findings, developmental status, laboratory test results, and imaging studies. Clinical information was obtained from the Hangzhou Children’s Social Welfare Center. Given the patient’s age and mild cognitive impairment, age-appropriate explanations about the medical examinations were provided by the caring clinicians and caregivers. A formal discussion regarding the specific genetic diagnosis was held with his legal guardians (institutional representatives).

### 2.3. Genetic studies

Genomic DNA was extracted from peripheral blood for sequencing analysis. Sequencing was performed on the Illumina platform, and primary data processing was conducted using the GATK software suite (Broad Institute, Cambridge). Sequencing reads were aligned to the University of California, Santa Cruz hg19 reference genome using the Burrows-Wheeler Aligner (BWA; Wellcome Trust Sanger Institute, Hinxton, UK). Variant annotation was performed with the Variant Effect Predictor tool, followed by filtering against specialized databases, including ClinVar, OMIM, hHman gene mutation database, and genome aggregation databaseg. Pathogenicity assessment and variant classification were determined through consensus among widely recognized computational prediction algorithms.

## 3. Case presentation

The patient was a 15-year-old adolescent boy from the Hangzhou Children’s Social Welfare Center. Birth history and parental anthropometric data were unavailable. He had a history of epilepsy and had been on long-term levetiracetam therapy without recurrence. His medical history did not include recurrent diarrhea or infections. The clinical phenotype was characterized by multiple dysmorphic features – particularly involving the face and digits – along with short stature and mild intellectual impairment.

**Figure 1.**
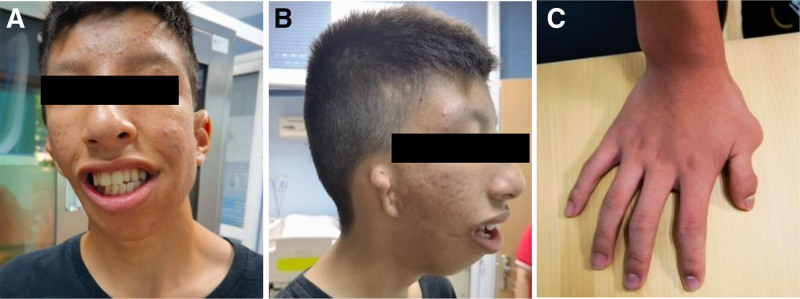
Photos of our patient. (A) Facial asymmetry, bulbous nose, broad nasal bridge, dental anomalies, micrognathia, and elongated skull shape. (B) Right ear lobular ptosis, low-set ears, and micrognathia. (C) Syndactyly of the right thumb.

Physical examination showed a height of 1.59 m (below the third percentile) and a weight of 42 kg. Cognitive impairment was mild, with no difficulty in everyday verbal communication. The patient responded accurately to questions, followed commands appropriately, and demonstrated normal motor function. No signs of autism spectrum disorder, attention-deficit/hyperactivity disorder, or compulsive behaviors were observed, and he exhibited neither anxiety nor insomnia. Dysmorphic features included facial asymmetry, a bulbous nose, a broad nasal bridge, right ear lobular ptosis, low-set ears, malocclusion, micrognathia, and an elongated skull shape (Fig. 1A and B). Additional findings included right thumb syndactyly (Fig. 1C), myopia, and amblyopia (in both eyes). No abnormalities were identified in the liver, spleen, or kidneys, and no hepatosplenomegaly or renal enlargement was observed. The clinical manifestations are summarized in Table 1.

**Table 1:** Clinical phenotypic characteristics.

Result	Depiction
Growth retardation	Height 1.59 m (<third percentile); weight 42 kg
Neurodevelopmental deficits	Mild cognitive impairment and a history of epilepsy (managed with levetiracetam)
Dysmorphic features and skeletal anomalies	Facial asymmetry, bulbous nose, broad nasal bridge, right ear lobular ptosis, low-set ears, malocclusion, micrognathia, elongated skull shape, and right thumb syndactyly
Ocular deficits	Myopia and amblyopia
Cardiac findings	Sinus bradycardia and mild mitral/tricuspid regurgitation

kg = kilogram, m = meter.

Routine laboratory tests, including hematological, urinary, fecal, and biochemical analyses, showed no clinically significant abnormalities. Electroencephalography revealed epileptiform discharges in both frontal regions, more prominent in the right frontal area, and electrocardiography demonstrated sinus bradycardia. Ultrasound examination of the liver, gallbladder, pancreas, spleen, and kidneys showed no abnormalities, and spinal radiograph revealed no deformities.

**Figure 2.**
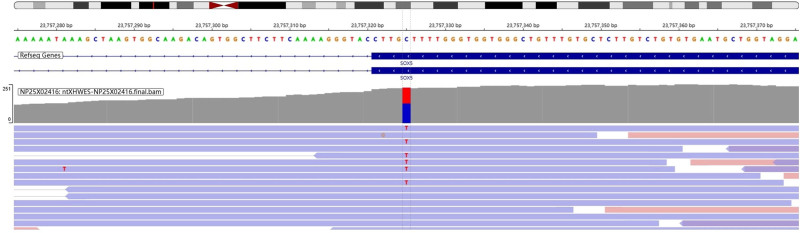
Whole-exome sequencing revealed a mutation of c.1160G>A in the *SOX5* gene. *SOX5* = SRY-related high-mobility group box 5.

Based on the above findings, a genetic cause was suspected, and genetic analysis was performed. Testing identified a heterozygous c.1160G>A variant in the coding region of the *SOX5* gene, located at 12p12.1. This mutation results in the substitution of serine with asparagine at amino acid position 387 (p.Ser387Asn, NM_006940.6) and was confirmed by whole-exome sequencing (Fig. 2). According to the American College of Medical Genetics and Genomics criteria, this missense variant is classified as a variant of uncertain significance. In light of the clinical presentation, a diagnosis of LAMSHF was established.

The patient continues to take levetiracetam for epilepsy and is receiving dental care for maxillofacial deformities.

## 4. Discussion

Mutations in *SOX5* have been widely reported to cause LAMSHF, which presents with diverse clinical manifestations such as developmental delay, dysmorphic features, hypotonia, and strabismus. This report contributes to expanding the understanding of the phenotypic spectrum and pathogenic *SOX5* variants by describing a Chinese adolescent with LAMSHF. The key findings include identification of a previously unreported de novo *SOX5* variant, observation of sinus bradycardia and dolichocephaly – features not previously described in this syndrome and of uncertain clinical significance, and recognition of phenotypic overlap with other neurodevelopmental disorders, which may complicate diagnosis.

### 4.1. Genetic findings and pathogenicity assessment

*SOX5* encodes a DNA-binding protein that recognizes oligonucleotides containing the consensus motif AACAAT.^[2]^ As a member of the group D *SOX* gene family, *SOX5* produces both short and long alternatively spliced transcripts that are expressed in multiple human tissues. The gene demonstrates spatiotemporal expression, with differential regulation across developmental stages, and is notably expressed in the adult testis and fetal brain.^[3,4]^ The long isoform, known as L-SOX5, functions downstream of *SOX9* and is expressed in chondrocyte condensations, where it plays an essential role in normal cartilage development.^[5]^ By contrast, the short isoform encodes a 43-kDa high-mobility group box protein with features similar to transcriptional activators and is specifically expressed in postmeiotic germ cells, with particularly high levels in round spermatids.^[2]^

Studies have shown that *SOX5* is essential for the migration of newly formed deep-layer neurons and contributes to the refinement of laminar-specific neuronal identities during postmigratory differentiation. Loss of *SOX5* expression disrupts the development of the neural crest, placode, and neural plate border. Furthermore, *SOX5* cooperates with *SOX6*, another member of the SOXD transcription factor family, to activate neural stem cells in a reversible quiescent state, thereby regulating and maintaining the neurogenic niche in the subgranular zone of the hippocampal dentate gyrus. In addition, *SOX5* acts in concert with the transcription factors *SOX6* and *SOX9*, forming the chondrogenic trio essential for chondrocyte proliferation and differentiation. These proteins remain active throughout the chondrocyte lineage, from the pre-mesenchymal to the pre-hypertrophic phase of the growth plate.^[6]^ Animal studies have demonstrated that homozygous deletion of the *SOX5* gene in mice results in lethal skeletal malformations and defects in deep-layer cortical projection neurons at birth, whereas heterozygous deletion produces a normal lifespan without apparent abnormalities.^[7]^ These findings suggest that *SOX5* mutations may impair neural and skeletal development through these mechanisms, leading to clinical features such as intellectual disability, epilepsy, and multiple congenital anomalies in affected children.

Reported *SOX5* gene variants include missense, nonsense, frameshift, and splice-site mutations.^[8]^ Genetic testing in the present case revealed a c.1160G>A missense variant in *SOX5* (12p12.1), resulting in the substitution of serine with asparagine at position 387 (p.Ser387Asn). This variant has not been previously documented. Although classified as a variant of uncertain significance according to the American College of Medical Genetics and Genomics criteria, its close correlation with the clinical features of the patient – intellectual disability, epilepsy, and specific malformations – strongly suggests pathogenic relevance.

### 4.2. Clinical manifestations

The symptoms of LAMSHF vary in severity and frequency.^[9,10]^ Developmental delay and intellectual disability are the most common features, reported in approximately 99% of cases, while facial dysmorphism and ocular abnormalities occur in more than 60% of affected individuals. The dysmorphic features were typically mild and variable, commonly including frontal bossing, down-slanting palpebral fissures, prominent philtral ridges, crowded teeth, and auricular anomalies.^[9]^ Several of these features were observed in the present case, including developmental delay, intellectual disability, facial asymmetry, a bulbous nose, a broad nasal bridge, malocclusion, right ear lobular ptosis, low-set ears, micrognathia, and right thumb syndactyly. Notably, facial dysmorphism in patients with LAMSHF may become more pronounced with age.^[11]^

Interestingly, epilepsy occurs in fewer than 25% of patients,^[6]^ and syndactyly is rarely reported. Among the published cases with epilepsy, electroencephalographic abnormalities typically involve the occipital, temporal, and parietal regions, differing from the prominent frontal lobe epileptiform discharges observed in our patient. This variation in localization may influence seizure characteristics, treatment approaches, and clinical outcomes.^[6,7,11]^

Beyond the manifestations listed in the OMIM database, this case presented with sinus bradycardia and dolichocephaly – features not previously described in association with this disorder. Whether these findings are directly attributable to the specific amino acid substitution remains uncertain and warrants further investigation. Conversely, several OMIM-reported features, including spinal deformities, dystonia, and stereotyped behaviors, were absent in this patient, highlighting the wide phenotypic variability of LAMSHF.

### 4.3. Diagnosis and differential diagnosis

Because LAMSHF presents with diverse and nonspecific clinical features, it must be differentiated from several other conditions, including short stature syndromes, Partington syndrome, Yuan–Harel–Lupski syndrome, X-linked intellectual disability with cerebellar hypoplasia, and infantile hypotonia with psychomotor retardation and characteristic facies 2 syndrome.^[12]^ This is particularly important in patients who present only with mild intellectual disability and subtle facial dysmorphism, as in the present case, as misdiagnosis may lead to unnecessary and potentially harmful growth hormone therapy, which carries an increased tumor risk.^[1]^ Therefore, genetic testing is essential to establish a definitive diagnosis in children presenting with developmental delay, intellectual disability, and dysmorphic facial features.

### 4.4. Treatment strategies

The management of LAMSHF primarily focuses on rehabilitation and symptomatic care.^[1]^ Epilepsy, when present, is typically drug-responsive, and many adult patients achieve seizure control. By contrast, behavioral abnormalities, including aggression, are common and may progressively worsen, often resulting in reduced independence.^[11]^ Although long-term therapeutic follow-up is unavailable for this case, early multidisciplinary intervention is strongly recommended. A comprehensive care team – comprising geneticists, psychologists, speech and physical therapists, ophthalmologists, and caregivers – can optimize developmental outcomes in affected children. In language rehabilitation, a structured 3-step approach may be adopted, emphasizing basic communication skills, pronunciation, and language acquisition and comprehension. Visual therapy classes can further enhance nonverbal and visuospatial abilities. Family and social support also play critical roles in improving adaptive functioning. For instance, parental counseling has been shown to strengthen the father–child relationship, promote independence in self-care activities, and increase participation in daily household activities and family life. Treatment efficacy can be systematically monitored using standardized tools such as the Stanford-Binet Intelligence Scale to assess cognitive ability and the Functional Scale of Social Maturity to evaluate adaptive behavior.^[13]^

The patient continues to take levetiracetam for epilepsy with good seizure control and no recurrence. He is also receiving dental care for maxillofacial deformities. Regular antiepileptic therapy is recommended to maintain seizure control, along with continued use of an orthotic device to improve facial contour and occlusal function, and optical correction when necessary. Ongoing electroencephalographic monitoring and periodic neurodevelopmental assessments are advised, together with targeted speech and behavioral rehabilitation therapies. Regular evaluation of growth parameters by an endocrinologist is also recommended to ensure appropriate developmental follow-up.

## 5. Limitations and suggestions

Despite our best efforts to refine this study, we must acknowledge its inherent limitations. The classification of the identified variant as one of uncertain significance is a key constraint, as we were unable to perform recommended confirmatory Sanger sequencing or trio analysis. This was due to significant logistical challenges, compounded by the patient’s status as an orphan under institutional care, which precluded obtaining additional biological samples for familial validation. Furthermore, the retrospective nature of this case report imposed restrictions on phenotypic characterization. The absence of Tanner staging for pubertal development, the reliance on clinical examination rather than formal quantitative tests for vision and hearing, and the use of behavioral observation rather than a standardized IQ test for cognitive assessment all represent gaps in quantitative data collection. To ensure accuracy, we have consequently described the cognitive observation using the behaviorally based term “mild cognitive impairment.”

Moving forward, future research would benefit greatly from prospective designs that prioritize obtaining necessary consents and samples for comprehensive genetic validation. Such studies should also mandate standardized phenotyping protocols, including Tanner staging, formal ophthalmologic and audiometric testing, and validated neurocognitive assessments, to build a more robust and quantifiable evidence base for genotype-phenotype correlations.

## 6. Conclusion

This case was diagnosed as LAMSHF caused by a previously unreported c.1160G>A missense variant in *SOX5* (12p12.1). The patient presented with characteristic features, including epilepsy, distinctive facial dysmorphism, intellectual disability, and skeletal anomalies. Notably, physical examination revealed sinus bradycardia and dolichocephaly – findings not previously documented in LAMSHF. Whether these cardiac features are related to the syndrome remains to be determined. Genetic testing confirmed the diagnosis of LAMSHF and excluded phenotypically overlapping disorders such as short stature syndromes, Partington syndrome, Yuan–Harel–Lupski syndrome, X-linked intellectual disability with cerebellar hypoplasia, and infantile hypotonia with psychomotor retardation and characteristic facies 2 syndrome, thereby preventing inappropriate interventions. Current treatment for LAMSHF focuses on symptom management. The patient continues to receive antiepileptic therapy with levetiracetam. To address intellectual disability and other developmental challenges, a multidisciplinary approach is recommended, integrating neurological follow-up, cognitive and language therapy, physical and occupational therapy, and psychosocial support, to enhance independence and adaptive functioning. This comprehensive strategy aims to optimize functional outcomes and improve quality of life and social integration.

## Acknowledgments

We sincerely thank the patient and his guardians for their cooperation and participation in this study.

## Author contributions

**Conceptualization:** Jingting Xu, Xiao Song, Linghui Zeng.

**Funding acquisition:** Chaochun Zou.

**Writing – review & editing:** Chaochun Zou.

**Writing – original draft:** Jingting Xu, Xiao Song, Linghui Zeng.
